# Manual and automated analysis of atrophy patterns in dementia with Lewy bodies on MRI

**DOI:** 10.1186/s12883-022-02642-0

**Published:** 2022-03-24

**Authors:** Eya Khadhraoui, Sebastian Johannes Müller, Niels Hansen, Christian Heiner Riedel, Philip Langer, Charles Timäeus, Jens Wiltfang, Caroline Bouter, Claudia Lange, Marielle Ernst

**Affiliations:** 1 Institute of Diagnostic and Interventional Neuroradiology, University Medical Center Göttingen (UMG), Georg-August-University, Göttingen, Germany; 2 Department of Psychiatry and Psychotherapy, University Medical Center Göttingen (UMG), Georg-August-University, Göttingen, Germany; 3 Department of Nuclear Medicine, University Medical Center Göttingen (UMG), Georg-August-University, Göttingen, Germany; 4 German Center for Neurodegenerative Diseases (DZNE), Göttingen, Germany; 5 Neurosciences and Signaling Group, Institute of Biomedicine (iBiMED), Department of Medical Sciences, University of Aveiro, Aveiro, Portugal

**Keywords:** Dementia with Lewy Bodies, MRI, Atrophy, Substantia innominate

## Abstract

**Background:**

Dementia with Lewy bodies (DLB) is the second most common dementia type in patients older than 65 years. Its atrophy patterns remain unknown. Its similarities to Parkinson's disease and differences from Alzheimer's disease are subjects of current research.

**Methods:**

The aim of our study was (i) to form a group of patients with DLB (and a control group) and create a 3D MRI data set (ii) to volumetrically analyze the entire brain in these groups, (iii) to evaluate visual and manual metric measurements of the innominate substance for real-time diagnosis, and (iv) to compare our groups and results with the latest literature. We identified 102 patients with diagnosed DLB in our psychiatric and neurophysiological archives. After exclusion, 63 patients with valid 3D data sets remained. We compared them with a control group of 25 patients of equal age and sex distribution. We evaluated the atrophy patterns in both (1) manually and (2) via Fast Surfers segmentation and volumetric calculations. Subgroup analyses were done of the CSF data and quality of 3D T1 data sets.

**Results:**

Concordant with the literature, we detected moderate, symmetric atrophy of the hippocampus, entorhinal cortex and amygdala, as well as asymmetric atrophy of the right parahippocampal gyrus in DLB. The caudate nucleus was unaffected in patients with DLB, while all the other measured territories were slightly too moderately atrophied. The area under the curve analysis of the left hippocampus volume ratio (< 3646mm^3^) revealed optimal 76% sensitivity and 100% specificity (followed by the right hippocampus and left amygdala). The substantia innominata’s visual score attained a 51% optimal sensitivity and 84% specificity, and the measured distance 51% optimal sensitivity and 68% specificity in differentiating DLB from our control group.

**Conclusions:**

In contrast to other studies, we observed a caudate nucleus sparing atrophy of the whole brain in patients with DLB. As the caudate nucleus is known to be the last survivor in dopamine-uptake, this could be the result of an overstimulation or compensation mechanism deserving further investigation. Its relative hypertrophy compared to all other brain regions could enable an imaging based identification of patients with DLB via automated segmentation and combined volumetric analysis of the hippocampus and amygdala.

**Supplementary Information:**

The online version contains supplementary material available at 10.1186/s12883-022-02642-0.

## Introduction

Dementia with Lewy bodies (DLB) is behind approximately 4% of all diagnosed dementias and in 7.5% of those in secondary care [1]. After Alzheimer’s disease (AD), it is the second most common dementia type in patients aged above 65 years [2]. Early diagnosis remains challenging. While the influence of cerebrospinal fluid (CSF), single photon emission computerized tomography (SPECT), positron emission tomography (PET) [3] and magnetic resonance imaging (MRI) diagnostics is increasing, clinical parameters are still the most important factors in making an accurate diagnosis [4].

The aim of our work is to analyze whether there is an imaging-based marker for DLB; on the one hand by means of a visual or manual metric score with the advantage of immediate application, on the other hand with more time-consuming volumetry.

Studies from Tokyo [5–7] analyzed the substantia innominata (SI) and reported an association between atrophy and DLB. The SI is a narrow area in the basal forebrain located below the globus pallidus at the level of the anterior commissure. It includes the nucleus basalis of Meynert. The SI is not yet included in the atlases of conventional segmentation algorithm.

In order to analyze the value of the SI as an imaging marker of DLB, we developed a new, pragmatic approach to measure the SI. Moreover, we performed an automated segmentation of the cerebral structures to assess their volume and to analyze specific atrophy pattern in DLB. We then compared these two methods in patients with DLB and a control group without neurological or psychiatric disease. We analyzed subgroups using CSF biomarkers to expose subgroups that are more affected.

## Methods

### Participant’s population

We analyzed patients diagnosed with clinically confirmed DLB following the *Fourth consensus report of the DLB Consortium* [4] and an MRI of the brain with a 3D T1 data sets from 01.01.2013 to 31.12.2020,. Since many patients had been diagnosed according to the old criteria [8], we carried out a retrospective evaluation according to the *Fourth consensus report of the DLB Consortium* [4].

The control group consisted of patients without neurological or psychiatric diseases and of a similar age and sex distribution. Exclusion criteria of the control group were a Fazekas score [9] of 2 or more and a global cortical atrophy (GCA) score [10] of 2 or more. We included only patients with a 3D T1 weighted data set of the same time interval as the DLB group (01.01.2013–31.12.2020). Patients were identified relying on the database search of our Picture Archiving and Communication System (PACS) system and analyzed by reading diagnostic findings and medical letters.

### MRI analysis

3D T1 data sets were acquired on two different MRI scanners (1.5 Tesla Siemens AvantoFit and 3.0 Tesla Siemens Magnetom/PrismaFit) between 01.01.2013 and 31.12.2020. We found two types of 3D data sets: favored T1 MP-RAGE (Magnetization Prepared—RApid Gradient Echo) and the faster T1 VIBE (Volumetric interpolated breath-hold examination) sequences for restless patients.

All subjects were scanned in sagittal orientation with a voxel resolution of 1.0 × 1.0 × 1.0 mm (parameters: MP-RAGE 1.5 T: scan time 298 s, TR 1.700 ms, TE 2.460 ms, flip angle 8°, TI 900 ms, 3.0 T: scan time 260 s, TR 2.000 ms, TE 2.980 ms, flip angle 9°, TI 900 ms, VIBE 1.5 T: scan time 225 s, TR 5.770 ms, TE 2.380 ms, flip angle 10°, 3.0 T: scan time 108 s, TR 4.960 ms, TE 2.240 ms, flip angle 9°).

We retrospectively analyzed MRIs of patients with DLB and of the control group by Fastsurfer and two independent raters blinded to the diagnosis. The raters were radiologists with 4 years (rater 1, EK) and 8 years (rater 2, ME) of neuroradiologic MRI experience imaging dementia.

### Automated volumetric MRI analysis

In the first step, we used the 3D Slicer (Version 4.10.2, https://www.slicer.org/) to transform from the DICOM (Digital Imaging and Communications in Medicine) to NIFTI (Neuroimaging Informatics Technology Initiative) file format. For segmentation, Fastsurfer [11] was used (Version commit dabf1e02e6253cac8bd3d641958b01e5348ea0e7, https://github.com/Deep-MI/FastSurfer/commit/dabf1e02e6253cac8bd3d641958b01e5348ea0e7) with the procedure call: run_fastsurfer.sh –fs_license $FREESURFER_HOME/license.txt –sd $out_path –sid $filename –t1 $f/$filename.nii –parallel –threads 24 –batch 64 –order 3 –vol_segstats. Surface statistics were obtained via FMRIB Software Library v6.0 (FSL 6.0), Version 6.0.4, from https://fsl.fmrib.ox.ac.uk/fsl/fslwiki/FslInstallation recon_surf). Used graphic card was GPU nVidia GV100, Driver 455.45.01, CUDA Version 11.1. Operating sytem was Ubuntu 18.04.5 LTS. Data from each patient’s stats folder was stored in a separate Excel-file. We applied the free/fast surfer’s standard segmentation algorithm (Fischl, 2012) and Desikan-Kiliany-Tourville DKTatlas.aseg.stats [12–14]. Each segmentation was controlled manually. For each segmentation, 38 base and white-matter stats and 62 cortex volumes were analyzed.

### Visual MRI analysis

**Fig. 1:**
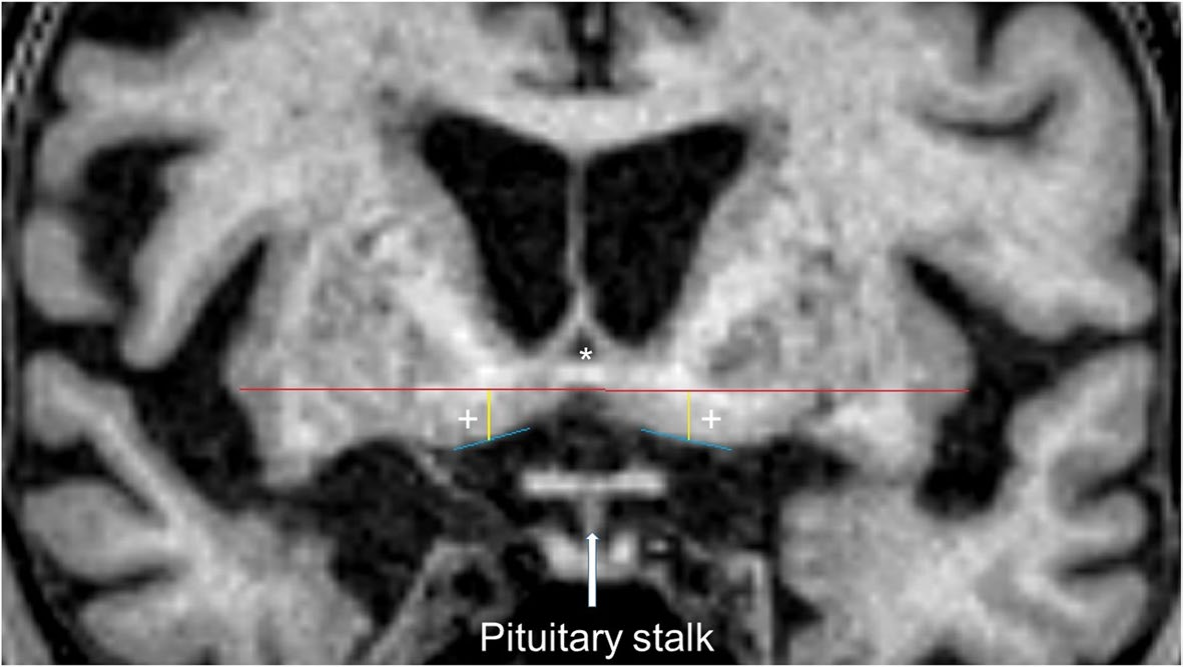
Measurement example. *: Anterior commissure; + : nucleus basalis Meynert; red line: horizontal line under the anterior commissure; blue line: line under the nucleus basalis Meynert; yellow line: distance

**Fig. 2:**
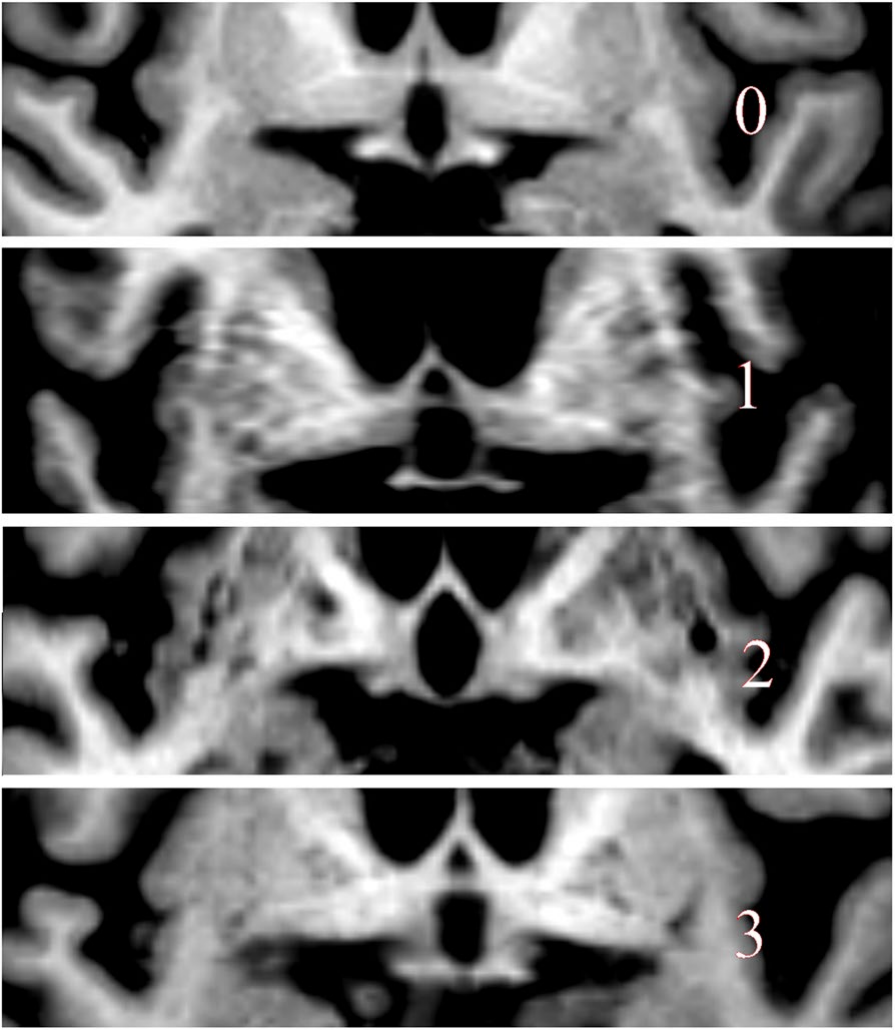
Atrophy score example.: Showing examples of the visual atrophy score. It should be mentioned that in case 3 no gray matter is detectable, even if the measured distance is normal

A coronal image was reconstructed from T1 3D sequences including the pituitary stalk and anterior commissure; see Fig. 1 for an example. We saved the reconstructed images and measurements in the PACS system for further analyses. We first assessed a visual analogue SI-score for the cortical thickness of the SI using a 4-step scale: 0 (no atrophy), 1 (mild atrophy), 2 (moderate atrophy), 3 (severe atrophy). Examples of atrophy scores are in Fig. 2.

### Manual metric MRI analysis

Since the interrater-reliability of visual ratings was low for sequences of poor quality, we added a quality-independent metric measurement, as in Fig. 1. (1) A horizontal line directly under the anterior commissure was drawn. (2) Two tangential lines to the base of the nucleus basalis of each hemisphere were drawn. (3) Two vertical lines from the anterior commissure line through the middle of each of the nucleus basalis lines were drawn, and that distance was measured.

### Cerebrospinal fluid diagnostics

Cerebrospinal fluid (CSF) samples via lumbar puncture were taken in 55 of the 63 patients with DLB. The Neurochemistry Laboratory of the Neurology Department (University Medical Center Göttingen) did CSF evaluation. CSF data contains total tau protein (t-tau, reference value < 450 pg/ml), phosphorylated tau protein 181 (ptau 181, reference value < 61 pg/ml), Beta-Amyloid 1–42 (reference value > 450 pg/ml), Beta-Amyloid 1–40 (reference value 8400–22,600 pg/ml) and Beta-Amyloid-Ratio 1–42/1–40 (10 * Beta-Amyloid-Ratio 1–42/1–40, reference value > 0.5, respectively 0.6).

### Statistical analysis

We used the Statistica, version 13 program (TIBCO Software Inc., Palo Alto, CALIFORNIA, USA). Significance level was set to *P* < 0.05. Interrater agreement was evaluated using Fleiss’ kappa [15] for the visual score and intraclass correlation coefficient (ICC) for the measurements [16]. We calculated the latter using the libraries in R Version 4: irr, readxl, lpSolve and psych.

Shapiro–Wilk (for *n* < 50) and Shapiro-Francia (for *n* >  = 50) [17–19] tests were used to test normal distribution. We used t-Test and Mann Whitney U test to evaluate significant differences in distribution or median. Correction for multiple comparisons was performed using the Holm-Bonferroni method [20].

### Subgroup analyses

We analyzed subgroup, (i) for the T1 sequence type (MP-RAGE and VIBE) to detect quality dependent errors, and (ii) for a Beta-Amyloid-Ratio 1–42/1–40 of 0.5, respectively 0.6 [21, 22], to expose potentially more affected subgroups and to detect any differences in LBD with and without AD comorbidity subgroup [23].

### Normalizations

Additional analysis with normalized volumes of estimated total intracranial volume (eTIV) and summarized segmented brain volumes (with/without the inner liquor system) were made.

## Results

### Participants

**Fig. 3:**
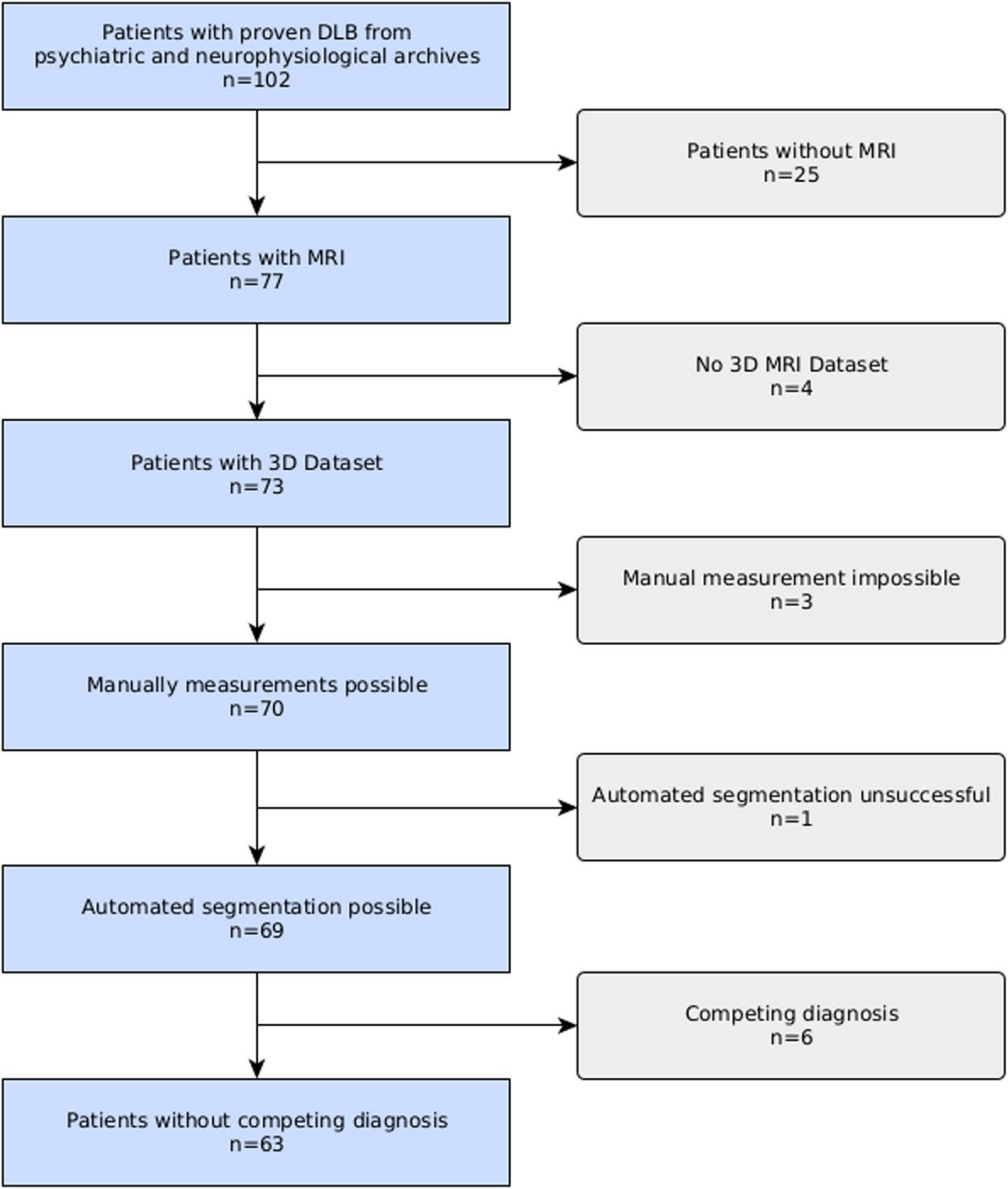
Flow chart DLB patients.: A flow chart of the inclusion of patients with Dementia with Lewy Bodies

We were able to confirm the diagnosis of DLB according to the *Fourth consensus report of the DLB Consortium* [4] in a group of 102 patients with DLB from our psychiatric and neurophysiological archives. We excluded 25 patients without an MRI. No 3D T1 data sets were available from four patients. Movement artifacts were too extreme for manual measurements in three cases, and in another, the automated segmentation malfunctioned due to movement artifacts. Six patients were excluded by competing diagnoses. See Fig. 3 for the associated flowchart. Our final database contained 63 patients (27 females) with diagnosed DLB, mean age (at time of MRI) 74.9 ± 7.0 (range 53–89 years). Our control group consisted of 25 patients (10 females) without neurological or psychiatric disease. Mean age (at time of MRI) was 74.8 ± 7.4 years (mean ± standard deviation, range 57–89 years). Sixteen of them had undergone MRI during staging (8 melanomas, 2 lymphomas, 2 breast cancers, 2 non-small cell lung cancers, 1 small cell lung cancer, and 1 urothelial carcinoma), two patients with lung sarcoidosis, and seven with various complaints. Detailed demographic and CSF data is available in Additional File 1.

### Sequences

We assessed 46 T1 MP-RAGE and 17 T1 VIBE images in the DLB group. MRI data sets of the control group contained 12 T1 MP-RAGE and 13 T1 VIBE sequences.

### Automated volumetric MRI analysis

Patients with DLB exhibited 10% average atrophy of the white and gray matter as well as symmetric, moderate atrophy of hippocampus and entorhinal cortex. The amygdala was more strongly affected (t-Test; *p* < 0.001 left and right). The asymmetric atrophy of the right parahippocampal gyrus we noted also deserves mention. Detailed volumetric data of all DLB patients is available in Additional File 2 and 3. Data on mesial temporal structures are illustrated in Table 1. We noticed the biggest differences (mean) between patients with DLB and the control group in the inferior lateral ventricle.

**Table 1:** Volumetric analysis of selected structures (See Supplementary for full statistics)

Structure	DLB		Control group		Diff
	**Mean mm**^**3**^	**SD mm**^**3**^	**Mean mm**^**3**^	**SD mm**^**3**^	
Left caudate nucleus	3210	767	2927	496	-9%
Right caudate nucleus	3062	766	2701	676	**-12%**
Left entorhinal cortex	1766	524	2043	406	**14%**
Right entorhinal cortex	1768	507	2020	356	**13%**
Left gyrus parahippocampalis	1649	370	1787	360	8%
Right gyrus parahippocampalis	1536	317	1768	292	**13%**
Left amygdala	1313	334	1605	266	**18%**
Right amygdala	1447	375	1761	258	**18%**
Left hippocampus	3292	487	3727	445	**12%**
Right hippocampus	3342	518	3748	437	**11%**
Left inferior lateral ventricle	1653	944	792	402	**-52%**
Right inferior lateral ventricle	1638	872	713	424	**-56%**

significant differences are marked in bold. Mixed T1 MP-RAGE and VIBE values, for single values see Supplemental Table 2 and 3

Interestingly, the left nucleus caudatus volume was smaller (3097 ± 503 mm^3^, mean ± standard deviation) in our control group than in DLB patients (3262 ± 662 mm^3^) without achieving significance (*t*-Test, *p* = 0.52).

### Visual MRI analysis

Our DLB group showed a mean SI-score-left of 0.99 ± 1.15 (mean ± standard deviation) and SI-score-right of 1.17 ± 1.09. Our control group achieved a mean SI-score-left of 0.35 ± 0.67 and SI-score-right of 0.7 ± 0.65. Mann–Whitney U test showed significant differences of the distribution of ratings for the left (*p* = 0.03), but not for the right (*p* = 0.15) side. Interrater-reliability was moderate (Fleiss’ kappa 0.48, *p* < 0.001, 2 raters).

### Manual metric MRI analysis

The DLB patients’ left sided SI-distances measured 0.63 ± 0.12 mm and the right sided SI-distances 0.59 ± 0.9 mm, the control groups left sided SI-distances measured 0.67 ± 0.11 mm and the right sided SI-distances 0.64 ± 0.9 mm. Shapiro-Francia test accepted the thesis for normal distribution. t-Test showed significant differences for the measurements between patients with DLB and the control group on the right hemisphere (right *p* = 0.02; left *p* = 0.12). Interrater-reliability was excellent with a single score ICC(A,1) of 0.889 with a 95%-confidence Interval of 0.819 to 0.928. Measurements on a standardized, already preformatted coronal image improved the single score ICC(A,1) to 0.979 (95%-confidence Interval of 0.969 to 0.986).

### Subgroup analysis

(i) **T1 MP-RAGE and T1 VIBE**
  Although our subgroup analyses for MRI grouping of the DLB and control group patients via T1 MP-RAGE and T1 VIBE data yielded no significant differences in the manual measurements, visual SI-scores (Mann–Whitney U test, *p* < 0.05) did differ significantly between MP-RAGE and VIBE. Our volumetric findings differed in measuring cortical structures. White matter and basal structures were of equal volume except for the right caudate nuclei, which was significantly (12%) smaller in patients with T1 VIBE than in patients with MP-RAGE (*t*-Test, *p* < 0.03 in DLB and *p* < 0.02 in the control group). The left caudate nucleus was not significantly smaller (5%, *t*-Test, *p* > 0.29 for both groups). In analyzing this error, we noticed that in approximately 20% of all our segmentations based on T1 VIBE sequences, the right caudate nucleus had not been completely delineated because of low contrast; in 5% of the segmentations, the left caudate nucleus was only partially included. Shapiro–Wilk accepted the thesis of a normal distribution of measured volumes for the subgroups MP-RAGE und VIBE, but not for both together. Hence, we made the ROC and AUC analyses in MP-RAGE patients only, as described below.
(ii) **CSF data**

An AD comorbidity in DLB occurs more often (approx. 25%) than in other dementias [24]. A selective lowering of Beta-Amyloid 1–42 (and of the ratio as well) indicates such an AD comorbidity. To detect any differences between patients suffering from DLB with and without AD, we divided the 55 DLB patients with available CSF data into two groups. Hence, the threshold for Beta-Amyloid 1–42 / Beta-Amyloid 1–40 ratio is still matter of debate and depends on the liquor labor; we tested a subgrouping for 0.5 and 0.6: (i) Beta-Amyloid 1–42 / Beta-Amyloid 1–40 ratio > 0.5 (true *n* = 45, false m = 10) and (ii) Beta-Amyloid 1–42 / Beta-Amyloid 1–40 ratio > 0.6 (true *n* = 39, false m = 16). Mean values and standard deviations did not significantly differ and separate Mann–Whitney U tests for these subgroups showed no significant differences for the visual SI-score, manual measurements and automated volumetric analyses (*p* > 0.1, MP-RAGE only).

### Differentiating DLB from controls

Youden’s J analyses of AUC estimated visual scores < 1 as being the optimal cut-off value with a 51% sensitivity and 84% specificity of the visual score for the left hemisphere (right side 49% sensitivity, 81% specificity). For the right hemisphere, an SI-distance of < 6 mm showed an optimized AUC with 51% optimal sensitivity and 68% specificity for SI measurements in differentiating DLB from the control group (left side 50% sensitivity, 64% specificity).

The most significant volumes for the differentiation of DLB and the control group after correction were the right lateral and right inferior lateral ventricle (*p* = 0.00001), followed by the left lateral and left inferior lateral ventricle (*p* = 0.00008 and *p* = 0.00016). However, we think that these volumes are not good markers, because of the overlaps with competing diseases (especially Alzheimer's disease). Other significant volumes after correction (Holm-Bonferroni method), were in order (p ascending): left hippocampus, cortex of right pars orbitalis, third ventricle, left and right amygdala, right hippocampus.

Since we observed significant atrophy of the amygdala and insignificant hypertrophy of the caudate nucleus, we combined them in a ratio (CN:AM) for a better differentiation from the control group and eventually other atrophy types. For further differentiation of AD patients, we also analyzed the ratio caudate nucleus/hippocampus (CN:HI).

A threshold for the left hippocampus volume of 3646 mm^3^ showed optimal 76% sensitivity and 100% specificity for the differentiation of DLB and the control group (right, 3600 mm^3^, 69% sensitivity and 100% specificity). A value of 1590 mm^3^ for the volume of the left amygdala revealed 85% sensitivity and 71% specificity (right, 1600 mm^3^, 71% sensitivity and 65% specificity). AUC analyses of the volume of the inferior lateral ventricle (threshold 900 mm^3^ for left and right side) showed a 77% (right 81%) sensitivity and 70% (both sides) specificity.

In order to pay attention to the observation of the caudate nucleus sparing atrophy, we also test several ratios including its volume. Area under the curve analysis of the left caudate nucleus volume/left amygdala volume (CN:AM left) ratio showed optimal 66% sensitivity and 86% specificity at a 2.25 cut-off. The *t*-Test revealed significant differences (mean DLB 2.55 ± 0.81 vs. mean control group 1.85 ± 0.36, *p* < 0.0001). The right CN:AM ratio showed an optimal 74% sensitivity and 71% specificity at a 1.87 cut-off. Left CN:HI demonstrated 85% sensitivity and 71% specificity at a 0.81 cut-off. Right CN:HI showed 50% sensitivity and 100% specificity at a 0.96 cut-off.

Hence, for the differentiation of DLB and control cohort, these ratios were not superior to the single volume threshold of hippocampus and amygdala.

### Normalizations

We could not find any significant influence of normalizations on our results. The lateral ventricle remains the most significant distinguisher. We discarded the use of eTIV, which was too imprecisely.

## Discussion

Our investigation of 63 patients with confirmed DLB and a 3D T1 database, showed slight to moderate atrophy of the whole brain with slightly more atrophy in the mesial temporal structures, especially the amygdala. We performed visual and manual analyses of the substantia innominata and showed a distinct atrophy, but not suitable for screening.

Our atrophy analysis including the amygdala in DLB patients confirms previous neuroimaging findings [25, 26]. Burton et al. showed a significant inverse correlation between amygdala volume in MRI and percent area of Lewy bodies in the amygdala [27].

The amygdala’s profound atrophy is associated with specific DLB neuropathology caused by Lewy bodies. Animal models have demonstrated behavioral dysfunction in conjunction with alpha synuclein inclusions in the amygdala [28], underpinning a potential role of the amygdala in our DLB patient group’s dysfunctional behavior and psychiatric symptoms.

We noticed that our visual SI-atrophy score should be exclusively evaluated by relying on T1 MP-RAGE sequences, while the more reliable manual measurements can be assessed using VIBE as well.

The manual measurement method our study demonstrates is a pragmatic and reliable approach for assessing SI atrophy; it even revealed slight differences in our DLB subgroups.

The two MRI analysis approaches revealed moderate atrophy of the SI in the DLB group. This is in accordance with neuropathological studies observing severe atrophy of the nucleus basalis of Meynert in DLB patients [29]. Early MRI analyses assume a variations in SI atrophy depending on the underlying disease [5]. While in AD the atrophy seems to correspond with the illness´ progression, the atrophy in DLB seems to be more pronounced [6]. Volumetric measurements suggest that the SI is also influenced by Parkinson’s disease (PD) [30]. Comparative volumetric analysis revealed more severe atrophy in PD and DLB than in AD [31].

Additional structural changes in the hippocampus occur in AD and DLB [32]. Although the DLB phenotype is known to depend on characteristic hippocampal atrophy [33], we detected no volumetric differences between the subgroups in our study. Consistent with a large study from the European DLB consortium [34], we observed homogeneous atrophy of the whole brain. In contrast to this study, there was one exception: the caudate nucleus was not atrophied. Thus, in our patient group, DLB presented as a caudate nucleus sparing atrophy. Even if volumetric measurements of the gray matter differed significantly between T1 MP-RAGE and T1 VIBE, we could not detect any significant caudate nucleus atrophy compared to the control group selected according to clinical characteristics as in other studies [35, 36]. This fits in well with the fact that severe limitations in motor skills and in everyday life were only occasionally documented.

Despite of FastSurfer, there are additional tools for the segmentations of the basal fore brain available, which should be evaluated in the future, e.g. from Zaborszky [37].

CSF data and regional brain atrophy seem to correlate in patients with DLB, especially beta-amyloid and medial temporal lobe atrophy [38]. We found no significant differences in patients with or without pathological liquor data, especially the Beta-Amyloid-Ratio 1–42/1–40, nor did we detect any correlations between volumetric data and CSF data in our small patient group. These results may be due to the fact that our patients present none of the relevant mixed pathologies influenced by AD pathology. The introduced volume ratios enable DLB patients to be clearly differentiated from control patients, and may also enable DLB to be differentiated from AD and PD—a subject to be addressed in future studies.

### Limitations

Measuring atrophy remains a challenging task that can be simplified and standardized via automated volumetric analysis. Nevertheless, we noted sequence-dependent differences which should always be included in analyses. It would make sense to always program the same T1 MP-RAGE sequence on the same MR scanner. However, this was not possible due to our study’s retrospective nature.

A final weakness of our study is that we formed our control group from an MRI data base consisting of hospital patients, however they were lacking neurological and psychiatric diseases.

## Conclusion

In our study, DLB patients exhibited slight to moderate whole brain atrophy with accentuation on the amygdala but an absent atrophy in the caudate nucleus.

We report on a pragmatic approach for taking reliable measurements of the SI which enables the rapid definition of the atrophy underlying DLB.

## Supplementary Information

**Additional file 1:** **Table S1.** Demographic and CSF data of patients with dementia with Lewybodies.

**Additional file 2:** **Table S2.** Manual measurements and Volumetric results using the Desikan–Killiany–Tourvilleatlas and FastSurfer for patients and control group with T1 MP-RAGE and T1 VIBEsequences.

**Additional file 3: Table S3. **Manual measurements and Volumetric results using the Desikan–Killiany–Tourvilleatlas and FastSurfer for patients and control group with T1 MP-RAGE sequences.

## Data Availability

The datasets used and analyzed during the current study are partially available in the additional files and on request. Individual data from patients were not published so that no identification can take place.

## Abbreviations

AD: Alzheimer’s disease
AUC: Area under curve
cMRI: Cranial magnetic resonance imaging
CSF: Cerebrospinal fluid
DLB: Dementia with Lewy bodies
DWI: Diffusion weighted imaging
ICC: Interclass correlation coefficient
MCI: Mild cognitive impairment
MR: Magnetic resonance
PD: Parkinson’s disease
ROC: Receiver operating characteristics
SI: Substantia innominata
SD: Standard deviation
