# Supplementary material for: Manual and automated analysis of atrophy patterns in dementia with Lewy bodies on MRI
Source: BMC Neurol. 2022 Mar 24;22:114. doi: 10.1186/s12883-022-02642-0 (PMC8943955; doi:10.1186/s12883-022-02642-0)
Supplement: Supplementary file 1 — Additional file 1: Table S1. Demographic and CSF data of patients with dementia with Lewybodies. [file 12883_2022_2642_MOESM1_ESM.docx]

**Manual and automated analysis of atrophy patterns in dementia with Lewy bodies on MRI**

Supplemental Table S1 – Demographic and CSF data of patients with dementia with Lewy bodies.

|  |  | **DLB patients** | | | | | **control group** | | | | |
| --- | --- | --- | --- | --- | --- | --- | --- | --- | --- | --- | --- |
| **Demographic data** | **Unit** | **n** | **mean** | **min** | **max** | **SD** | **n** | **mean** | **min** | **max** | **SD** |
| Age | years | 63 | 74.9 | 53 | 89 | 7.0 | 25 | 74.8 | 57 | 89 | 7.4 |
| Education | (0-3) | 21 | 1.5 | 1 | 3 | 0.8 | 0 |  |  |  |  |
| Disease duration | years | 23 | 2.8 | 0.2 | 8.0 | 2.4 | 0 |  |  |  |  |
| MMSE | (0-30) | 40 | 21.1 | 4 | 30 | 6.4 | 0 |  |  |  |  |
| **Liquor data** |  |  |  |  |  |  |  |  |  |  |  |
| Tau | pg/ml | 55 | 399 | 75 | 953 | 219 | 0 |  |  |  |  |
| Phospho-Tau 181 P | pg/ml | 55 | 60 | 15 | 122 | 26 | 0 |  |  |  |  |
| Beta-Amyloid 1-42 | pg/ml | 55 | 875 | 359 | 2072 | 342 | 0 |  |  |  |  |
| Beta-Amyloid 1-42 / Beta Amyloid 1-40 ratio | - | 55 | 0.9 | 0.2 | 2.0 | 0.4 | 0 |  |  |  |  |

**Supplemental Table S1 – Legend**: CSF – cerebrospinal fluid, Education – (0 – visited no school, 1 – secondary school to grade 9 / “Hauptschule”, 2 – secondary school to grade 10 / “Realschule”, 3 – Secondary to grade 12/13 / “Gymnasium”), MMSE – Mini Mental State Examination.
