# Supplementary material for: Manual and automated analysis of atrophy patterns in dementia with Lewy bodies on MRI
Source: BMC Neurol. 2022 Mar 24;22:114. doi: 10.1186/s12883-022-02642-0 (PMC8943955; doi:10.1186/s12883-022-02642-0)
Supplement: Supplementary file 2 — Additional file 2: Table S2. Manual measurements and Volumetric results using the Desikan–Killiany–Tourvilleatlas and FastSurfer for patients and control group with T1 MP-RAGE and T1 VIBEsequences. [file 12883_2022_2642_MOESM2_ESM.docx]

**Manual and automated analysis of atrophy patterns in dementia with Lewy bodies on MRI**

Supplemental Table S2 –Manual measurements and Volumetric results using the Desikan–Killiany–Tourville atlas and FastSurfer for patients and control group with T1 MP-RAGE and T1 VIBE sequences.

|  |  | **DLB patients** | | | | | **control group** | | | | |
| --- | --- | --- | --- | --- | --- | --- | --- | --- | --- | --- | --- |
| **Attribute** | **Unit** | **n** | **mean** | **min** | **max** | **SD** | **n** | **mean** | **min** | **max** | **SD** |
| Visual Score SI left | (0-3) | 63 | 0.97 | 0.00 | 3.00 | 1.16 | 25 | 0.40 | 0.00 | 2.00 | 0.71 |
| Visual Score SI right | (0-3) | 63 | 1.14 | 0.00 | 3.00 | 1.09 | 25 | 0.72 | 0.00 | 2.00 | 0.68 |
| Measured distance left | mm | 63 | 0.63 | 0.36 | 0.95 | 0.12 | 25 | 0.66 | 0.55 | 0.95 | 0.10 |
| Measured distance right | mm | 63 | 0.59 | 0.34 | 0.75 | 0.09 | 25 | 0.63 | 0.50 | 0.82 | 0.09 |
| Measured distance sum | mm | 63 | 1.22 | 0.80 | 1.63 | 0.17 | 25 | 1.30 | 1.08 | 1.65 | 0.16 |
| Left-Cerebral-White-Matter | mm³ | 63 | 234302 | 161946 | 333573 | 39695 | 25 | 253613 | 181865 | 327777 | 36788 |
| Left-Lateral-Ventricle | mm³ | 63 | 27364 | 6697 | 64327 | 11841 | 25 | 15733 | 6826 | 35371 | 7708 |
| Left-Inf-Lat-Vent | mm³ | 63 | 1653 | 521 | 4191 | 944 | 25 | 792 | 294 | 1594 | 402 |
| Left-Cerebellum-White-Matter | mm³ | 63 | 13758 | 6620 | 18966 | 2168 | 25 | 15220 | 11110 | 18176 | 2106 |
| Left-Cerebellum-Cortex | mm³ | 63 | 51618 | 39605 | 72055 | 6849 | 25 | 56244 | 47131 | 63731 | 5020 |
| Left-Thalamus | mm³ | 63 | 5870 | 4011 | 9232 | 936 | 25 | 6438 | 5084 | 8004 | 728 |
| Left-Caudate | mm³ | 63 | 3210 | 1045 | 4985 | 767 | 25 | 2927 | 2134 | 3882 | 496 |
| Left-Putamen | mm³ | 63 | 3856 | 782 | 5624 | 876 | 25 | 4077 | 2768 | 5513 | 645 |
| Left-Pallidum | mm³ | 63 | 1819 | 613 | 3151 | 363 | 25 | 1929 | 1493 | 2627 | 318 |
| 3rd-Ventricle | mm³ | 63 | 2296 | 1291 | 3916 | 632 | 25 | 1778 | 843 | 3209 | 573 |
| 4th-Ventricle | mm³ | 63 | 2018 | 947 | 3659 | 656 | 25 | 1733 | 1008 | 2794 | 592 |
| Brain-Stem | mm³ | 63 | 20200 | 15018 | 26759 | 2522 | 25 | 20951 | 17083 | 25654 | 2421 |
| Left-Hippocampus | mm³ | 63 | 3292 | 2392 | 4177 | 487 | 25 | 3727 | 2599 | 4571 | 445 |
| Left-Amygdala | mm³ | 63 | 1313 | 562 | 2085 | 334 | 25 | 1605 | 1090 | 2308 | 266 |
| CSF | mm³ | 63 | 1607 | 821 | 2843 | 426 | 25 | 1356 | 766 | 1991 | 314 |
| Left-Accumbens-area | mm³ | 63 | 290 | 0 | 548 | 138 | 25 | 360 | 119 | 590 | 138 |
| Left-VentralDC | mm³ | 63 | 3842 | 2681 | 5358 | 521 | 25 | 4111 | 3463 | 5347 | 469 |
| Left-choroid-plexus | mm³ | 63 | 1089 | 288 | 1571 | 250 | 25 | 1028 | 647 | 1312 | 208 |
| Right-Cerebral-White-Matter | mm³ | 63 | 232808 | 161547 | 323135 | 38337 | 25 | 250184 | 182633 | 317668 | 36379 |
| Right-Lateral-Ventricle | mm³ | 63 | 25607 | 7495 | 62151 | 10902 | 25 | 13888 | 6076 | 26160 | 6382 |
| Right-Inf-Lat-Vent | mm³ | 63 | 1638 | 446 | 4138 | 872 | 25 | 713 | 284 | 1990 | 424 |
| Right-Cerebellum-White-Matter | mm³ | 63 | 13431 | 4592 | 19462 | 2441 | 25 | 14386 | 9458 | 17500 | 1997 |
| Right-Cerebellum-Cortex | mm³ | 63 | 52133 | 40249 | 67157 | 6304 | 25 | 56543 | 48152 | 66581 | 4912 |
| Right-Thalamus | mm³ | 63 | 5755 | 3215 | 9139 | 1024 | 25 | 6444 | 4982 | 7727 | 778 |
| Right-Caudate | mm³ | 63 | 3062 | 1435 | 5089 | 766 | 25 | 2701 | 1540 | 3775 | 676 |
| Right-Putamen | mm³ | 63 | 3806 | 1275 | 5499 | 877 | 25 | 4026 | 2850 | 5374 | 581 |
| Right-Pallidum | mm³ | 63 | 1799 | 483 | 3166 | 407 | 25 | 1841 | 1487 | 2348 | 241 |
| Right-Hippocampus | mm³ | 63 | 3342 | 2106 | 4497 | 518 | 25 | 3748 | 2555 | 4388 | 437 |
| Right-Amygdala | mm³ | 63 | 1447 | 516 | 2270 | 375 | 25 | 1761 | 1376 | 2515 | 258 |
| Right-Accumbens-area | mm³ | 63 | 336 | 0 | 576 | 132 | 25 | 398 | 159 | 599 | 127 |
| Right-VentralDC | mm³ | 63 | 3856 | 2917 | 5380 | 546 | 25 | 4065 | 3349 | 5295 | 495 |
| Right-choroid-plexus | mm³ | 63 | 1135 | 268 | 1865 | 282 | 25 | 1047 | 625 | 1457 | 226 |
| WM-hypointensities | mm³ | 63 | 5884 | 1005 | 24552 | 4932 | 25 | 2174 | 799 | 6108 | 1265 |
| CC_Posterior | mm³ | 63 | 927 | 158 | 1367 | 205 | 25 | 946 | 270 | 1298 | 218 |
| CC_Mid_Posterior | mm³ | 63 | 541 | 128 | 927 | 152 | 25 | 627 | 409 | 843 | 116 |
| CC_Central | mm³ | 63 | 699 | 127 | 1174 | 216 | 25 | 820 | 475 | 1142 | 179 |
| CC_Mid_Anterior | mm³ | 63 | 610 | 114 | 1320 | 285 | 25 | 748 | 354 | 1238 | 259 |
| CC_Anterior | mm³ | 63 | 940 | 254 | 1431 | 217 | 25 | 938 | 624 | 1575 | 211 |
| ctx-lh-caudalanteriorcingulate | mm³ | 63 | 2338 | 400 | 3238 | 506 | 25 | 2434 | 1728 | 3068 | 418 |
| ctx-lh-caudalmiddlefrontal | mm³ | 63 | 4712 | 1339 | 8210 | 1472 | 25 | 4368 | 1508 | 7726 | 1617 |
| ctx-lh-cuneus | mm³ | 63 | 2745 | 1386 | 3873 | 430 | 25 | 2752 | 1757 | 4026 | 542 |
| ctx-lh-entorhinal | mm³ | 63 | 1766 | 90 | 2628 | 524 | 25 | 2043 | 1006 | 2653 | 406 |
| ctx-lh-fusiform | mm³ | 63 | 5914 | 1620 | 10066 | 1297 | 25 | 6595 | 3905 | 9162 | 1138 |
| ctx-lh-inferiorparietal | mm³ | 63 | 9071 | 4414 | 13483 | 1777 | 25 | 9407 | 5405 | 13625 | 2227 |
| ctx-lh-inferiortemporal | mm³ | 63 | 9904 | 5503 | 14607 | 1765 | 25 | 10740 | 6262 | 12899 | 1682 |
| ctx-lh-isthmuscingulate | mm³ | 63 | 1988 | 984 | 3326 | 407 | 25 | 2147 | 1565 | 2896 | 368 |
| ctx-lh-lateraloccipital | mm³ | 63 | 10334 | 5279 | 14393 | 1619 | 25 | 11000 | 6943 | 15053 | 1831 |
| ctx-lh-lateralorbitofrontal | mm³ | 63 | 6768 | 3218 | 9321 | 1022 | 25 | 7179 | 4995 | 8929 | 1021 |
| ctx-lh-lingual | mm³ | 63 | 4729 | 415 | 7328 | 947 | 25 | 5196 | 4023 | 6625 | 682 |
| ctx-lh-medialorbitofrontal | mm³ | 63 | 3566 | 1958 | 5194 | 617 | 25 | 3820 | 2818 | 4955 | 493 |
| ctx-lh-middletemporal | mm³ | 63 | 10830 | 7456 | 16258 | 1967 | 25 | 12218 | 7949 | 16712 | 2031 |
| ctx-lh-parahippocampal | mm³ | 63 | 1649 | 554 | 2438 | 370 | 25 | 1787 | 1139 | 2449 | 360 |
| ctx-lh-paracentral | mm³ | 63 | 3172 | 1748 | 4755 | 635 | 25 | 3002 | 2105 | 4055 | 616 |
| ctx-lh-parsopercularis | mm³ | 63 | 3115 | 1631 | 4786 | 643 | 25 | 3195 | 2035 | 4493 | 754 |
| ctx-lh-parsorbitalis | mm³ | 63 | 1492 | 264 | 2230 | 375 | 25 | 1579 | 870 | 2535 | 373 |
| ctx-lh-parstriangularis | mm³ | 63 | 3216 | 1422 | 5238 | 666 | 25 | 3372 | 1823 | 4490 | 688 |
| ctx-lh-pericalcarine | mm³ | 63 | 1373 | 626 | 2129 | 347 | 25 | 1405 | 977 | 2174 | 345 |
| ctx-lh-postcentral | mm³ | 63 | 7973 | 4895 | 11634 | 1355 | 25 | 8231 | 6013 | 10119 | 1330 |
| ctx-lh-posteriorcingulate | mm³ | 63 | 2616 | 1469 | 3721 | 442 | 25 | 2813 | 1999 | 3366 | 358 |
| ctx-lh-precentral | mm³ | 63 | 10268 | 5625 | 14638 | 1728 | 25 | 10299 | 6462 | 13861 | 1872 |
| ctx-lh-precuneus | mm³ | 63 | 6638 | 3867 | 9119 | 1078 | 25 | 7026 | 5083 | 10844 | 1334 |
| ctx-lh-rostralanteriorcingulate | mm³ | 63 | 2810 | 1532 | 4271 | 573 | 25 | 3083 | 2089 | 4547 | 596 |
| ctx-lh-rostralmiddlefrontal | mm³ | 63 | 8347 | 5175 | 11764 | 1568 | 25 | 8543 | 5274 | 11469 | 1793 |
| ctx-lh-superiorfrontal | mm³ | 63 | 17871 | 9415 | 26384 | 3444 | 25 | 17437 | 10822 | 24424 | 3365 |
| ctx-lh-superiorparietal | mm³ | 63 | 7550 | 3020 | 11296 | 1447 | 25 | 7766 | 5376 | 11891 | 1834 |
| ctx-lh-superiortemporal | mm³ | 63 | 12956 | 6171 | 18376 | 2237 | 25 | 14344 | 8821 | 19429 | 2407 |
| ctx-lh-supramarginal | mm³ | 63 | 7553 | 3084 | 10749 | 1371 | 25 | 7791 | 4554 | 10416 | 1302 |
| ctx-lh-transversetemporal | mm³ | 63 | 754 | 224 | 1280 | 185 | 25 | 808 | 473 | 1113 | 185 |
| ctx-lh-insula | mm³ | 63 | 4847 | 2013 | 7214 | 939 | 25 | 4861 | 2963 | 7020 | 827 |
| ctx-rh-caudalanteriorcingulate | mm³ | 63 | 1677 | 360 | 2544 | 448 | 25 | 1780 | 1126 | 2834 | 397 |
| ctx-rh-caudalmiddlefrontal | mm³ | 63 | 4200 | 927 | 7091 | 1223 | 25 | 3827 | 1631 | 6875 | 1457 |
| ctx-rh-cuneus | mm³ | 63 | 2515 | 1213 | 3961 | 524 | 25 | 2718 | 1741 | 3917 | 530 |
| ctx-rh-entorhinal | mm³ | 63 | 1768 | 156 | 2784 | 507 | 25 | 2020 | 1245 | 2543 | 356 |
| ctx-rh-fusiform | mm³ | 63 | 5967 | 2286 | 9278 | 1357 | 25 | 6569 | 4195 | 9726 | 1199 |
| ctx-rh-inferiorparietal | mm³ | 63 | 9999 | 3396 | 13643 | 1956 | 25 | 10896 | 6170 | 14882 | 2221 |
| ctx-rh-inferiortemporal | mm³ | 63 | 9858 | 4893 | 14208 | 1954 | 25 | 11023 | 6849 | 14301 | 1803 |
| ctx-rh-isthmuscingulate | mm³ | 63 | 1875 | 1189 | 3067 | 370 | 25 | 2079 | 1617 | 3262 | 406 |
| ctx-rh-lateraloccipital | mm³ | 63 | 10276 | 5305 | 15217 | 1792 | 25 | 11111 | 8088 | 13967 | 1692 |
| ctx-rh-lateralorbitofrontal | mm³ | 63 | 7161 | 4141 | 9700 | 1037 | 25 | 7509 | 5205 | 8924 | 899 |
| ctx-rh-lingual | mm³ | 63 | 4849 | 2463 | 6856 | 869 | 25 | 5397 | 3441 | 6954 | 801 |
| ctx-rh-medialorbitofrontal | mm³ | 63 | 3498 | 2106 | 5260 | 504 | 25 | 3736 | 2562 | 4869 | 521 |
| ctx-rh-middletemporal | mm³ | 63 | 10844 | 5759 | 15982 | 2122 | 25 | 12059 | 8211 | 15191 | 1870 |
| ctx-rh-parahippocampal | mm³ | 63 | 1536 | 412 | 2077 | 317 | 25 | 1768 | 1055 | 2516 | 292 |
| ctx-rh-paracentral | mm³ | 63 | 3139 | 1471 | 4602 | 640 | 25 | 3173 | 2248 | 4576 | 587 |
| ctx-rh-parsopercularis | mm³ | 63 | 3022 | 869 | 4276 | 631 | 25 | 3308 | 2038 | 4556 | 740 |
| ctx-rh-parsorbitalis | mm³ | 63 | 1576 | 678 | 2362 | 329 | 25 | 1888 | 1089 | 2580 | 396 |
| ctx-rh-parstriangularis | mm³ | 63 | 2927 | 1289 | 4508 | 714 | 25 | 3129 | 1888 | 4559 | 659 |
| ctx-rh-pericalcarine | mm³ | 63 | 1536 | 786 | 2453 | 355 | 25 | 1672 | 960 | 2379 | 324 |
| ctx-rh-postcentral | mm³ | 63 | 7536 | 3117 | 11375 | 1461 | 25 | 7599 | 5371 | 9805 | 1245 |
| ctx-rh-posteriorcingulate | mm³ | 63 | 2636 | 1466 | 3776 | 480 | 25 | 2827 | 2043 | 3280 | 354 |
| ctx-rh-precentral | mm³ | 63 | 9974 | 5294 | 14531 | 1686 | 25 | 10379 | 6862 | 13336 | 1710 |
| ctx-rh-precuneus | mm³ | 63 | 6895 | 3462 | 9909 | 1148 | 25 | 7525 | 5351 | 10596 | 1298 |
| ctx-rh-rostralanteriorcingulate | mm³ | 63 | 2010 | 998 | 3261 | 462 | 25 | 2017 | 1309 | 3243 | 484 |
| ctx-rh-rostralmiddlefrontal | mm³ | 63 | 8426 | 3439 | 13033 | 1875 | 25 | 8798 | 5295 | 11739 | 1838 |
| ctx-rh-superiorfrontal | mm³ | 63 | 19713 | 10572 | 28126 | 3707 | 25 | 19148 | 12065 | 24743 | 3573 |
| ctx-rh-superiorparietal | mm³ | 63 | 7361 | 1446 | 12126 | 1767 | 25 | 7777 | 4447 | 11845 | 2046 |
| ctx-rh-superiortemporal | mm³ | 63 | 12679 | 6024 | 16768 | 2188 | 25 | 13450 | 9234 | 17983 | 2148 |
| ctx-rh-supramarginal | mm³ | 63 | 6648 | 2424 | 9605 | 1415 | 25 | 7273 | 3796 | 9228 | 1462 |
| ctx-rh-transversetemporal | mm³ | 63 | 558 | 167 | 917 | 143 | 25 | 574 | 313 | 890 | 170 |
| ctx-rh-insula | mm³ | 63 | 4986 | 2307 | 6724 | 840 | 25 | 4949 | 2681 | 6303 | 780 |

**Supplemental Table S2 – Legend:** SI - substantia innominata, DLB - Dementia with Lewy bodies, SD - standard deviation, min – minimum, max –maximum, MP-RAGE - magnetization-prepared rapid gradient-echo, VIBE - volumetric interpolated breath-hold examination.
