# Supplementary material for: Manual and automated analysis of atrophy patterns in dementia with Lewy bodies on MRI
Source: BMC Neurol. 2022 Mar 24;22:114. doi: 10.1186/s12883-022-02642-0 (PMC8943955; doi:10.1186/s12883-022-02642-0)
Supplement: Supplementary file 3 — Additional file 3: Table S3. Manual measurements and Volumetric results using the Desikan–Killiany–Tourvilleatlas and FastSurfer for patients and control group with T1 MP-RAGE sequences. [file 12883_2022_2642_MOESM3_ESM.docx]

**Manual and automated analysis of atrophy patterns in dementia with Lewy bodies on MRI**

Supplemental Table S3 –Manual measurements and Volumetric results using the Desikan–Killiany–Tourville atlas and FastSurfer for patients and control group with T1 MP-RAGE sequences.

|  |  | **DLB patients** | | | | | **control group** | | | | |
| --- | --- | --- | --- | --- | --- | --- | --- | --- | --- | --- | --- |
| **Attribute** | **Unit** | **n** | **mean** | **min** | **max** | **SD** | **n** | **mean** | **min** | **max** | **SD** |
| Visual Score SI left | (0-3) | 46 | 0.85 | 0.00 | 3.00 | 1.09 | 12 | 0.33 | 0.00 | 1.00 | 0.52 |
| Visual Score SI right | (0-3) | 46 | 1.00 | 0.00 | 3.00 | 1.07 | 12 | 0.67 | 0.00 | 1.00 | 0.52 |
| Measured distance left | mm | 46 | 0.63 | 0.36 | 0.95 | 0.13 | 12 | 0.67 | 0.57 | 0.83 | 0.10 |
| Measured distance right | mm | 46 | 0.59 | 0.39 | 0.75 | 0.08 | 12 | 0.66 | 0.52 | 0.82 | 0.12 |
| Measured distance sum | mm | 46 | 1.22 | 0.80 | 1.63 | 0.17 | 12 | 1.33 | 1.09 | 1.65 | 0.20 |
| Left-Cerebral-White-Matter | mm³ | 46 | 226479 | 161946 | 333573 | 37455 | 12 | 239771 | 181865 | 297641 | 39426 |
| Left-Lateral-Ventricle | mm³ | 46 | 26813 | 6697 | 56046 | 11063 | 12 | 12485 | 6826 | 17800 | 4925 |
| Left-Inf-Lat-Vent | mm³ | 46 | 1661 | 521 | 4191 | 943 | 12 | 516 | 354 | 740 | 171 |
| Left-Cerebellum-White-Matter | mm³ | 46 | 13721 | 10684 | 18276 | 1836 | 12 | 15318 | 12255 | 17729 | 2169 |
| Left-Cerebellum-Cortex | mm³ | 46 | 50643 | 39605 | 71173 | 6168 | 12 | 55650 | 47131 | 63731 | 6349 |
| Left-Thalamus | mm³ | 46 | 5728 | 4313 | 7835 | 790 | 12 | 6625 | 5215 | 7490 | 846 |
| Left-Caudate | mm³ | 46 | 3262 | 2078 | 4985 | 662 | 12 | 3118 | 2377 | 3882 | 548 |
| Left-Putamen | mm³ | 46 | 4000 | 2681 | 5624 | 763 | 12 | 4468 | 4125 | 5196 | 385 |
| Left-Pallidum | mm³ | 46 | 1836 | 1339 | 3151 | 323 | 12 | 2079 | 1730 | 2627 | 358 |
| 3rd-Ventricle | mm³ | 46 | 2255 | 1291 | 3444 | 599 | 12 | 1716 | 1042 | 2589 | 610 |
| 4th-Ventricle | mm³ | 46 | 2025 | 999 | 3659 | 713 | 12 | 1962 | 1091 | 2592 | 536 |
| Brain-Stem | mm³ | 46 | 20094 | 15018 | 26151 | 2343 | 12 | 22233 | 17996 | 25654 | 2988 |
| Left-Hippocampus | mm³ | 46 | 3287 | 2392 | 4177 | 484 | 12 | 3933 | 3647 | 4101 | 171 |
| Left-Amygdala | mm³ | 46 | 1303 | 610 | 2085 | 322 | 12 | 1621 | 1351 | 1969 | 239 |
| CSF | mm³ | 46 | 1581 | 1027 | 2843 | 434 | 12 | 1165 | 797 | 1411 | 266 |
| Left-Accumbens-area | mm³ | 46 | 301 | 7 | 526 | 129 | 12 | 471 | 341 | 590 | 89 |
| Left-VentralDC | mm³ | 46 | 3820 | 2993 | 5358 | 490 | 12 | 4322 | 3668 | 5136 | 538 |
| Left-choroid-plexus | mm³ | 46 | 1105 | 659 | 1571 | 179 | 12 | 985 | 698 | 1312 | 243 |
| Right-Cerebral-White-Matter | mm³ | 46 | 226242 | 161547 | 323135 | 36470 | 12 | 239356 | 182633 | 294477 | 38444 |
| Right-Lateral-Ventricle | mm³ | 46 | 24587 | 7495 | 48131 | 9140 | 12 | 11441 | 6551 | 18848 | 4564 |
| Right-Inf-Lat-Vent | mm³ | 46 | 1649 | 571 | 4138 | 868 | 12 | 466 | 354 | 645 | 114 |
| Right-Cerebellum-White-Matter | mm³ | 46 | 13533 | 10462 | 19462 | 2036 | 12 | 14877 | 11804 | 17500 | 2279 |
| Right-Cerebellum-Cortex | mm³ | 46 | 51762 | 40249 | 67157 | 6010 | 12 | 56220 | 48152 | 66581 | 6840 |
| Right-Thalamus | mm³ | 46 | 5627 | 3215 | 7711 | 876 | 12 | 6537 | 5139 | 7519 | 814 |
| Right-Caudate | mm³ | 46 | 3176 | 2027 | 5089 | 650 | 12 | 3239 | 2552 | 3775 | 471 |
| Right-Putamen | mm³ | 46 | 3908 | 2473 | 5499 | 724 | 12 | 4476 | 4094 | 5374 | 466 |
| Right-Pallidum | mm³ | 46 | 1789 | 948 | 3166 | 353 | 12 | 1899 | 1578 | 2348 | 332 |
| Right-Hippocampus | mm³ | 46 | 3374 | 2361 | 4497 | 494 | 12 | 3888 | 3633 | 4253 | 257 |
| Right-Amygdala | mm³ | 46 | 1438 | 638 | 2270 | 349 | 12 | 1685 | 1376 | 2052 | 258 |
| Right-Accumbens-area | mm³ | 46 | 352 | 94 | 570 | 116 | 12 | 492 | 447 | 585 | 60 |
| Right-VentralDC | mm³ | 46 | 3824 | 2917 | 5380 | 534 | 12 | 4329 | 3425 | 5295 | 616 |
| Right-choroid-plexus | mm³ | 46 | 1193 | 722 | 1865 | 234 | 12 | 1006 | 685 | 1180 | 202 |
| WM-hypointensities | mm³ | 46 | 5666 | 1005 | 19594 | 4635 | 12 | 1663 | 1081 | 3063 | 740 |
| CC_Posterior | mm³ | 46 | 939 | 589 | 1367 | 170 | 12 | 980 | 771 | 1197 | 155 |
| CC_Mid_Posterior | mm³ | 46 | 510 | 242 | 927 | 137 | 12 | 668 | 491 | 826 | 137 |
| CC_Central | mm³ | 46 | 660 | 335 | 1174 | 201 | 12 | 755 | 557 | 995 | 193 |
| CC_Mid_Anterior | mm³ | 46 | 544 | 277 | 1320 | 247 | 12 | 649 | 354 | 1043 | 313 |
| CC_Anterior | mm³ | 46 | 933 | 584 | 1431 | 206 | 12 | 880 | 772 | 1039 | 94 |
| ctx-lh-caudalanteriorcingulate | mm³ | 46 | 2435 | 1662 | 3238 | 421 | 12 | 2420 | 1873 | 2864 | 439 |
| ctx-lh-caudalmiddlefrontal | mm³ | 46 | 5144 | 2346 | 8210 | 1212 | 12 | 6336 | 4508 | 7726 | 1322 |
| ctx-lh-cuneus | mm³ | 46 | 2821 | 2058 | 3873 | 380 | 12 | 3268 | 2645 | 4026 | 514 |
| ctx-lh-entorhinal | mm³ | 46 | 1817 | 472 | 2628 | 472 | 12 | 2392 | 2162 | 2567 | 157 |
| ctx-lh-fusiform | mm³ | 46 | 6119 | 3814 | 10066 | 1160 | 12 | 7484 | 5660 | 9162 | 1184 |
| ctx-lh-inferiorparietal | mm³ | 46 | 9363 | 6818 | 13483 | 1654 | 12 | 10738 | 8123 | 13394 | 1764 |
| ctx-lh-inferiortemporal | mm³ | 46 | 10021 | 6799 | 13652 | 1634 | 12 | 11525 | 10236 | 12899 | 1061 |
| ctx-lh-isthmuscingulate | mm³ | 46 | 2082 | 1461 | 3326 | 375 | 12 | 2378 | 1743 | 2896 | 467 |
| ctx-lh-lateraloccipital | mm³ | 46 | 10460 | 6277 | 14393 | 1567 | 12 | 12001 | 8678 | 14386 | 2003 |
| ctx-lh-lateralorbitofrontal | mm³ | 46 | 6795 | 5070 | 8577 | 922 | 12 | 7816 | 6301 | 8929 | 973 |
| ctx-lh-lingual | mm³ | 46 | 4913 | 3040 | 7328 | 734 | 12 | 5811 | 4670 | 6625 | 635 |
| ctx-lh-medialorbitofrontal | mm³ | 46 | 3494 | 1958 | 4940 | 556 | 12 | 3932 | 3479 | 4302 | 350 |
| ctx-lh-middletemporal | mm³ | 46 | 10867 | 7456 | 16258 | 2020 | 12 | 12589 | 11384 | 16712 | 2051 |
| ctx-lh-parahippocampal | mm³ | 46 | 1723 | 957 | 2438 | 333 | 12 | 2096 | 1865 | 2449 | 240 |
| ctx-lh-paracentral | mm³ | 46 | 3388 | 2337 | 4755 | 544 | 12 | 3598 | 3127 | 4055 | 383 |
| ctx-lh-parsopercularis | mm³ | 46 | 3245 | 2265 | 4786 | 579 | 12 | 3865 | 3222 | 4412 | 509 |
| ctx-lh-parsorbitalis | mm³ | 46 | 1579 | 874 | 2230 | 323 | 12 | 2008 | 1584 | 2535 | 331 |
| ctx-lh-parstriangularis | mm³ | 46 | 3223 | 2311 | 5238 | 590 | 12 | 3397 | 2886 | 4401 | 526 |
| ctx-lh-pericalcarine | mm³ | 46 | 1408 | 690 | 2129 | 343 | 12 | 1762 | 1341 | 2027 | 295 |
| ctx-lh-postcentral | mm³ | 46 | 8258 | 6204 | 11634 | 1255 | 12 | 9022 | 6891 | 10119 | 1281 |
| ctx-lh-posteriorcingulate | mm³ | 46 | 2647 | 1884 | 3721 | 438 | 12 | 2718 | 2183 | 3119 | 424 |
| ctx-lh-precentral | mm³ | 46 | 10750 | 8391 | 14638 | 1459 | 12 | 11803 | 9655 | 13861 | 1670 |
| ctx-lh-precuneus | mm³ | 46 | 6863 | 5368 | 9119 | 956 | 12 | 7604 | 5794 | 10844 | 1762 |
| ctx-lh-rostralanteriorcingulate | mm³ | 46 | 2759 | 1534 | 3696 | 478 | 12 | 2858 | 2089 | 3457 | 533 |
| ctx-lh-rostralmiddlefrontal | mm³ | 46 | 8607 | 6409 | 11764 | 1359 | 12 | 9473 | 7208 | 11469 | 1888 |
| ctx-lh-superiorfrontal | mm³ | 46 | 18820 | 13087 | 26384 | 2773 | 12 | 19750 | 17410 | 22610 | 1810 |
| ctx-lh-superiorparietal | mm³ | 46 | 7796 | 4658 | 10651 | 1185 | 12 | 9408 | 7657 | 11891 | 1509 |
| ctx-lh-superiortemporal | mm³ | 46 | 13083 | 9063 | 18376 | 1933 | 12 | 15063 | 12238 | 17569 | 1823 |
| ctx-lh-supramarginal | mm³ | 46 | 7819 | 5523 | 10749 | 1235 | 12 | 9006 | 7660 | 10416 | 965 |
| ctx-lh-transversetemporal | mm³ | 46 | 761 | 430 | 1268 | 155 | 12 | 846 | 641 | 1030 | 178 |
| ctx-lh-insula | mm³ | 46 | 5064 | 3636 | 7214 | 762 | 12 | 5127 | 4446 | 5463 | 361 |
| ctx-rh-caudalanteriorcingulate | mm³ | 46 | 1727 | 675 | 2544 | 425 | 12 | 1828 | 1338 | 2372 | 397 |
| ctx-rh-caudalmiddlefrontal | mm³ | 46 | 4538 | 2149 | 7091 | 949 | 12 | 5415 | 3921 | 6875 | 1229 |
| ctx-rh-cuneus | mm³ | 46 | 2589 | 1520 | 3961 | 497 | 12 | 3281 | 2786 | 3917 | 392 |
| ctx-rh-entorhinal | mm³ | 46 | 1823 | 727 | 2784 | 461 | 12 | 2219 | 1952 | 2422 | 188 |
| ctx-rh-fusiform | mm³ | 46 | 6040 | 3113 | 9278 | 1162 | 12 | 7292 | 5758 | 9726 | 1351 |
| ctx-rh-inferiorparietal | mm³ | 46 | 10202 | 6364 | 13643 | 1760 | 12 | 12291 | 10472 | 14882 | 1580 |
| ctx-rh-inferiortemporal | mm³ | 46 | 9943 | 5915 | 14208 | 1811 | 12 | 11855 | 9964 | 13532 | 1383 |
| ctx-rh-isthmuscingulate | mm³ | 46 | 1923 | 1232 | 3067 | 374 | 12 | 2214 | 1725 | 2539 | 376 |
| ctx-rh-lateraloccipital | mm³ | 46 | 10314 | 5305 | 15217 | 1721 | 12 | 12212 | 10230 | 13433 | 1191 |
| ctx-rh-lateralorbitofrontal | mm³ | 46 | 7141 | 5660 | 9499 | 909 | 12 | 7903 | 6572 | 8924 | 959 |
| ctx-rh-lingual | mm³ | 46 | 4939 | 3507 | 6856 | 808 | 12 | 5877 | 4654 | 6954 | 771 |
| ctx-rh-medialorbitofrontal | mm³ | 46 | 3524 | 2746 | 4581 | 408 | 12 | 3942 | 3305 | 4435 | 477 |
| ctx-rh-middletemporal | mm³ | 46 | 10871 | 7460 | 15982 | 1942 | 12 | 12187 | 10477 | 15191 | 1671 |
| ctx-rh-parahippocampal | mm³ | 46 | 1591 | 1011 | 2077 | 283 | 12 | 1988 | 1748 | 2516 | 277 |
| ctx-rh-paracentral | mm³ | 46 | 3339 | 2421 | 4602 | 550 | 12 | 3315 | 2672 | 3687 | 359 |
| ctx-rh-parsopercularis | mm³ | 46 | 3091 | 2128 | 4276 | 507 | 12 | 3730 | 2887 | 4411 | 587 |
| ctx-rh-parsorbitalis | mm³ | 46 | 1594 | 895 | 2362 | 323 | 12 | 2008 | 1703 | 2329 | 232 |
| ctx-rh-parstriangularis | mm³ | 46 | 2881 | 1762 | 4211 | 604 | 12 | 3182 | 2494 | 4377 | 630 |
| ctx-rh-pericalcarine | mm³ | 46 | 1538 | 786 | 2261 | 344 | 12 | 1860 | 1503 | 2166 | 268 |
| ctx-rh-postcentral | mm³ | 46 | 7786 | 5244 | 11375 | 1315 | 12 | 8474 | 6927 | 9805 | 1082 |
| ctx-rh-posteriorcingulate | mm³ | 46 | 2692 | 1837 | 3776 | 473 | 12 | 2780 | 2329 | 3280 | 384 |
| ctx-rh-precentral | mm³ | 46 | 10314 | 8031 | 14531 | 1405 | 12 | 11175 | 9367 | 12739 | 1407 |
| ctx-rh-precuneus | mm³ | 46 | 7067 | 5124 | 9909 | 1049 | 12 | 8133 | 6621 | 10596 | 1531 |
| ctx-rh-rostralanteriorcingulate | mm³ | 46 | 2008 | 1348 | 3261 | 422 | 12 | 1782 | 1309 | 2822 | 557 |
| ctx-rh-rostralmiddlefrontal | mm³ | 46 | 8768 | 5994 | 13033 | 1638 | 12 | 9269 | 7031 | 11539 | 1708 |
| ctx-rh-superiorfrontal | mm³ | 46 | 20617 | 14466 | 28126 | 3176 | 12 | 21871 | 18269 | 24665 | 2255 |
| ctx-rh-superiorparietal | mm³ | 46 | 7667 | 3704 | 12126 | 1523 | 12 | 9381 | 7458 | 11845 | 1674 |
| ctx-rh-superiortemporal | mm³ | 46 | 12775 | 9217 | 16768 | 2001 | 12 | 13387 | 10617 | 16114 | 2079 |
| ctx-rh-supramarginal | mm³ | 46 | 6879 | 4442 | 9605 | 1208 | 12 | 8401 | 7429 | 9228 | 700 |
| ctx-rh-transversetemporal | mm³ | 46 | 578 | 304 | 917 | 132 | 12 | 699 | 555 | 890 | 130 |
| ctx-rh-insula | mm³ | 46 | 5113 | 4046 | 6724 | 674 | 12 | 5263 | 4639 | 5778 | 443 |

**Supplemental Table S3 – Legend**: SI - substantia innominata, DLB - Dementia with Lewy bodies, SD - standard deviation, min – minimum, max –maximum, MP-RAGE - magnetization-prepared rapid gradient-echo.
